# Comparative Serum and Brain Pharmacokinetics of Quercetin after Oral and Nasal Administration to Rats as Lyophilized Complexes with β-Cyclodextrin Derivatives and Their Blends with Mannitol/Lecithin Microparticles

**DOI:** 10.3390/pharmaceutics15082036

**Published:** 2023-07-28

**Authors:** Konstantina Manta, Paraskevi Papakyriakopoulou, Anna Nikolidaki, Evangelos Balafas, Nikolaos Kostomitsopoulos, Sabrina Banella, Gaia Colombo, Georgia Valsami

**Affiliations:** 1 Department of Pharmacy, School of Health Sciences, National and Kapodistrian University of Athens, 15784 Athens, Greece; konstantina.manta@yahoo.gr (K.M.); ppapakyr@pharm.uoa.gr (P.P.); an_nikolidaki@hotmail.com (A.N.); 2 Laboratory Animal Facility, Centre of Clinical, Experimental Surgery and Translational Research, Biomedical Research Foundation of the Academy of Athens, 11527 Athens, Greece; vbalafas@bioacademy.gr (E.B.); nkostom@bioacademy.gr (N.K.); 3 Department of Life Sciences and Biotechnology, University of Ferrara, 44121 Ferrara, Italy; bnlsrn@unife.it (S.B.); clmgai@unife.it (G.C.)

**Keywords:** quercetin, cyclodextrins, pharmacokinetics, nose-to-brain transport, nasal powders, Alzheimer’s disease

## Abstract

Quercetin (Que) is one of the most studied flavonoids with strong antioxidant properties ascribed to its ability to bind free radicals and inactivate them. However, the low solubility of the compound along with its inadequate absorption after oral administration limit its beneficial effects. Que’s complexation with two different cyclodextrin (CD) derivatives (hydroxypropyl-β-CD and methyl-β-CD) via the neutralization/lyophilization method has been found to improve its physicochemical properties. Moreover, blends of the lyophilized powders with mannitol/lecithin microparticles (MLMPs) have been proposed as candidates for intranasal (IN) administration after in vitro and ex vivo evaluations. In this context, a comparative pharmacokinetic (PK) study of the IN vs oral administration of Que lyophilized powders and their blends with MLMPs (75:25 *w*/*w*) was performed on Wistar rats. The PK parameters estimated by a non-compartmental analysis using the sparse data methodology in Phoenix^®^ 8.3 (Certara, Princeton, NJ, USA) illustrated the effectiveness of IN administration either in brain targeting or in reaching the bloodstream. Significant levels of the compound were achieved at both sites, compared to those after oral delivery which were negligible. These results favor the potential application of the prepared Que nasal powders for systemic and nose-to-brain delivery for the prevention and/or treatment of neuroinflammatory degenerative conditions, such as Parkinson’s and Alzheimer’s disease.

## 1. Introduction

Many studies have pointed that oxidative stress is a major factor for the onset and progression of neurodegenerative disorders, including Alzheimer’s disease (AD), as well as pathological conditions of the brain [1,2,3]. More specifically, AD is characterized by the gradual degeneration of neurons, leading to the loss of cognitive ability, memory impairment and possible dysfunction in daily activities [4]. Free-radical-induced oxidative stress is strongly associated with all the main hypotheses reported for the development of AD, namely: (a) the β-amyloid accumulation hypothesis, (b) the Tau hypothesis, (c) the cholinergic hypothesis, (d) the stimulatory toxicity hypothesis and (e) the mitochondrial cascade hypothesis [1,3,5]. Therefore, research to identify molecules that bind and/or inactivate free radicals may be of great importance in the treatment of AD.

Quercetin (Que) is a natural flavonoid that is abundant in many fruits, vegetables and medicinal plants, characterized by strong antioxidant and anti-inflammatory activities [6,7,8]. Que interacts with free radicals and forms chelate complexes with metal ions, thus effectively inhibiting oxidative stress and inflammation which contribute to the appearance and progression of AD [9,10,11]. However, studies have shown that the activity of Que is limited by its low oral bioavailability [12]. Low water solubility and limited intestinal absorption are the main reasons for Que’s low bioavailability after per os administration [13,14]. Moreover, Que undergoes extensive first-pass metabolism by intestine and liver [14]. Nevertheless, the primary metabolites of Que in serum and the brain, i.e., glucurono-sulfo conjugates, also possess antioxidant and anti-inflammatory properties, as well as neuroprotective activity [15,16,17,18]. Several approaches for enhancing Que solubility have been reported in the literature, including the formation of inclusion complexes with cyclodextrin (CD) derivatives, as well as its formulation into amorphous solid or emulsion/nanoemulsion systems [19,20,21,22]. Hydrogels, cocrystals and amorphous solid dispersions have been also evaluated for improving Que systemic bioavailability using rodent animal models [23]. However, limited studies are reported for brain targeting and the increase in Que bioavailability at this site. Recently, shellac/caseinate-based nanocarriers were found to improve both the brain’s and systemic bioavailability of Que in Wistar rats [24]. Moreover, superparamagnetic iron oxide nanoparticles have successfully enhanced Que brain exposure in healthy rats [25]. Furthermore, nanoformulations have been developed as candidates for the nose-to-brain (NTB) administration of Que [26,27] and more extensively evaluated in the context of in vivo studies [28,29]. However, despite the advantages of powders compared to nanoformulations, in terms of stability, simplicity of production, cost and ease of administration, the technology of nasal powders has not been applied in Que NTB delivery.

To address the challenges of Que’s low oral bioavailability and insufficient penetration of the blood–brain barrier (BBB), the nasal route has emerged as a potential alternative for administration. This approach aims to overcome these limitations and improve Que’s access to the central nervous system (CNS). In this context, we have analyzed and characterized the nasal powders of Que used in previous studies [21,22,23,24,25,26,27,28,29,30]. More specifically, to increase Que’s aqueous solubility, lyophilized products were produced using the well-known host molecules hydroxypropyl-β-cyclodextrin (HP-β-CD) and methyl-β-cyclodextrin (Me-β-CD) as solubility enhancers [30,31]. These lyophilizates (Que-HP-β-CD and Que-Me-β-CD) were then blended with mannitol/lecithin microparticles (MLMPs) at various ratios to produce powders for nasal administration. Que’s in vitro diffusion across an artificial membrane and ex vivo permeation through rabbit nasal mucosa were evaluated for both the lyophilized products and their blends using Franz-type diffusion cells [31,32].

The present study intended to prove the NTB delivery of Que after IN administration, either in the form of lyophilizates with β-CDs derivatives (Que-HP-β-CD and Que-Me-β-CD) or as blends of the lyophilizates with MLMPs, in comparison to their oral administration. To this end, Wistar rats were used as animal models, and the serum and brain levels of Que were measured at predefined time points after administration. In order to assess the IN administration of Que for brain targeting, various PK parameters such as the AUC (area under the concentration–time curve), C_max_ (maximum observed concentration) and t_max_ (time of maximum concentration) were determined. Additionally, the specific indexes [33,34] were calculated to evaluate the extent of the direct NTB delivery of Que.

## 2. Materials and Methods

### 2.1. Materials

Quercetin (Que, MW: 302.24 g/mol), Methyl-β-cyclodextrin (Me-β-CD, MW: 1310 g/mol) and Hydroxypropyl-β-cyclodextrin (HP-β-CD, MW: 1460 g/mol) were purchased from Sigma-Aldrich (St. Louis, MO, USA), Fluka Chemika (Buchs, Switzerland) and Ashland (Covington, KY, USA), respectively. Naringenin was supplied from Alfa Aesar (Ward Hill, MA, USA). Mannitol (Ph. Eur.) was obtained from Lisapharma S.p.A. (Erba (CO), Italy), and soybean lecithin (Lipoid^®^ S45) was obtained from Lipoid AG (Steinhausen, Switzerland). Potassium dihydrogen phosphate and sodium acetate were acquired from Merck KGaA (Darmstadt, Germany), while sodium hydroxide (1 mol/L) was acquired from Chem-Lab NV (Zedelgem, Belgium). Glacial acetic acid was purchased from PanReac AppliChem (Chicago, IL, USA). Sodium acetate buffer solution (pH 5.0) was prepared using sodium acetate and glacial acetic acid in HPLC-grade water. The β-glucuronidase enzyme solution (3000 units/mL) was freshly prepared using the sodium acetate buffer. HPLC-grade solvents (water, methanol, acetonitrile) and reagents were obtained from Fischer Scientific (Pittsburgh, PA, USA). Triple-deionized water purchased from Fischer Scientific was used for all preparations.

### 2.2. Preparation of Que-CD Formulations

#### 2.2.1. Que-CD Lyophilizates

Que-Me-β-CD and Que-HP-β-CD lyophilized powders were prepared following the method described by Manta et al. (2020) [31]. Que was added to Me-β-CD and HP-β-CD water solutions under continuous stirring and light protection. The flavonoid/CD molar ratios were 1:1 and 1:2, respectively. A solution of 6% ammonium hydroxide was gradually added until Que dissolved completely, while monitoring and adjusting the pH to around 9–9.5. The obtained solution was then freeze-dried using a vacuum freeze dryer (BK-FD10T, Biobase Biodustry Co., Ltd. (Jinan, China)). Que content in the lyophilized powders was quantified using the method described by Papakyriakopoulou et al. (2021) [32].

#### 2.2.2. Que-Lyophilizate Solutions for Oral Gavage Administration

To prepare the solutions for the oral administration of Que at the desired doses, weighed amounts of Que-Me-β-CD or Que-HP-β-CD lyophilizates were dissolved in water for injection (WFI) to obtain concentrations of 1.66 mg/mL (Que dose: 0.83 mg, 2.5 mg/kg) and 0.29 mg/mL (Que dose: 0.145 mg, 0.45 mg/kg), respectively. A volume of 0.5 mL of the resulting solution was administered to each animal using the oral gavage technique. The solutions were prepared on the day of administration to ensure the stability of the compound.

#### 2.2.3. Spray-Dried MLMPs

MLMPs were prepared by spray-drying a mannitol/lecithin solution (with a ratio of 92:8 *w*/*w*) in ethanol following Balducci et al.’s method (2013) with the same operation settings for the Mini Spray Dryer B-191 (BÜCHI Labortechnik AG, Flawil, Switzerland) [35]. The spray-dried mannitol/lecithin powder was blended with the Que-Me-β-CD and Que-HP-β-CD complexes.

#### 2.2.4. Que-CD-MLMP Blends

Spray-dried MLMP powder was manually blended with Que-Me-β-CD and Que-HP-β-CD lyophilizates in glass vials at a 25:75 ratio (MPLPs: Que lyophilizates). The blends were mixed for 20 min and characterized through various analyses [32]. The quantification of Que’s content was determined by HPLC analysis, as described in the following section, Section 2.5.

### 2.3. Animal Experiments

#### 2.3.1. Animals and Housing Conditions

All animal experiments were performed in the animal facility of the Centre of Clinical, Experimental Surgery and Translational Research of the Biomedical Research Foundation of the Academy of Athens. The facility is registered as “breeding” and “experimental” facility according to the Greek Presidential Decree 56/2013, under the international legislation of European Community Directive 2010/63 on the Protection of Animals used for Experimental and Other Scientific Purposes (European Union, 2010). Wistar-type rats were used in the study and were housed in individually ventilated cages (Techniplast, Varese, Italy) under specific pathogen-free conditions and constant environmental conditions (12:12 h light:dark cycle, temperature of 22 ± 2 °C, relative humidity of 45 ± 10%). The rats were fed on irradiated pellets (2918 Teklad Global 18% Protein Rodent Diet, Harlan Laboratories, Indianapolis, IN, USA) and had access to tap water ad libitum. The cage bedding comprised corncob granules (REHOFIX^®^, J. Rettenmaier & Söhne Co., Germany, Rosenberg). Cages and bedding were changed once a week. All rats in the facility were screened regularly according to a health-monitoring program, complying with the Federation of European Laboratory Animal Science Association’s recommendations. The experimental protocol of the study was approved by the Veterinary Authorities of Region of Athens, Greece (Ethical approval num. 912432/Date of approval: 25 November 2020). The study was conducted in accordance with the ARRIVE guidelines and associated guidelines under EU Directive 2010/63/EU for animal experiments [36].

#### 2.3.2. Intranasal Administration

The Unidose Powder System (UDS; Aptar, Louveciennes, France) was used to administer the powder (https://www.aptar.com/products/pharmaceutical/unidose-nasal-powder-device-manufacturer/, accessed on 21 June 2023). It is a pre-metered single-dose powder insufflator device. The device comprises a mechanical pump connected to a nasal adapter, featuring a special tip designed for small animals and a reservoir for the solid formulation. Before administration, the insufflator’s reservoir was filled with 20 mg of lyophilizate or blend powder and accurately weighed, and then the device was assembled according to the manufacturer’s directions. Each loaded device was weighed before and after the actuation to determine the quantity of powder administered.

#### 2.3.3. In Vivo Study Dosing and Sampling Protocol

Εight-week-old Wistar-type rats (322 ± 52 g) were randomly divided into six groups. The animals in each group received a different treatment, namely: (a) Per os (PO) Que-Me-β-CD group (40 rats) and (b) PO Que-HP-β-CD group (21 rats) which received 0.5 mL of the respective lyophilizate solution. Groups (a, b) were further divided in 7 subgroups (3 animals per group), with each subgroup representing one sampling time point (15, 30, 45, 60, 120, 240 and 360 min after the treatment); lyophilizate groups (c,d) were IN Que-Me-β-CD (18 rats) and IN Que-HP-β-CD (18 rats); and blend groups were (e,f) IN Que-Me-β-CD:MLMPs (75:25) (18 rats) and IN Que-HP-β-CD:MLMPs (75:25) (18 rats). Groups (c–f) were further divided in 6 subgroups (3 animals per group), with each subgroup representing one sampling time point (5, 15, 30, 45, 60 and 90 min after the treatment). The mean IN dose of each formulation resulted by the mean emitted dose delivered after the actuation of the device. The doses of all tested formulations are listed in Table 1. Moreover, for the oral administration of lyophilizates (Que-Me-β-CD and Que-HP-β-CD), a dose equal to the intranasal one was selected. IN dose was administered to anaesthetized rats. Anesthesia was induced by intraperitoneal injection of ketamine (dose: 100 mg/kg, Ketamidor, Richter Pharma, Austria) and xylazine (dose: 0.1 mg/kg, Rompun, Bayer, Germany). The oral administration was performed by gavage. For powder administration, the technique described by Tiozzo Fasiolo et al. (2021) [34] was adopted. Briefly, the insufflator’s tip was inserted 1–2 mm into the nostril, and the powder was administered in a single shot by activating the pump. The device was then reweighed to determine the administered dose. At the predefined time points, each animal was sacrificed to collect blood and brain samples. Blood samples were taken via puncture of the lateral vesicular vein and then transferred into non-heparinized Eppendorf^®^ tubes (Hamburg, Germany) and centrifuged (10,000 rpm, 15 min, 4 °C) to separate serum. The brain was collected after total body perfusion with cold PBS (phosphate buffered saline, pH 7.4; KCl 0.2 g/L; NaCl 8 g/L; Na_2_HPO_4_ 1.15 g/L; KH_2_PO_4_ 0.2 g/L; 5 min, 120 mL) to remove the residual blood. For this purpose, the procedure described by Papakyriakopoulou et al. (2023) [37] was applied after being adjusted for rats. In detail, the PBS was perfused at a rate of 24 mL/min by means of a syringe pump (PLUS SEP-12S) connected with a 21G butterfly needle inserted into the heart’s left ventricle. After perfusion, the brain was gently dissected from the skull and weighed. In the case of IN group, the olfactory bulb was isolated and weighed for quantifying the drug inside, separate from the rest of the brain. Serum, brain and olfactory samples were frozen and stored at −70 °C until extraction and HPLC analysis.

### 2.4. Que Extraction from Biological Samples

The procedure to extract Que from the biological samples was adapted from Papakyriakopoulou et al. (2023) [26], using naringenin as internal standard.

#### 2.4.1. Parent-Que Extraction from Serum

A 40 μL serum sample was mixed with 25 μL of internal standard solution (naringenin 0.4 μg/mL in methanol), 10 μL of methanol and 5 μL HClO_4_ (14%) and then vortexed for 10 s. After the protein precipitation, the mixture was separated by centrifugation at 10,000 rpm, 4 °C for 10 min followed by the injection of an aliquot (30 μL) of the supernatant into the HPLC system for analysis. The same procedure was applied for the preparation of the blank serum samples spiked with Que standard solutions used for the calibration curves.

#### 2.4.2. Parent-Que Extraction from Rat Brain and Olfactory Bulb

On the day of analysis, each brain sample was homogenized using a T10 ULTRA-TURRAX^®^ (IKA Werke, Staufen im Breisgau, Germany) in the presence of WFI (tissue:WFI ratio of 1:1 *w*/*w*). For each olfactory bulb, the tissue was homogenized with a disposable polypropylene pestle (Sigma-Aldrich, St. Louis, MO, USA) in a 2 mL Eppendorf^®^ microtube [34]. To determine Que in tissue samples, 40 μL of homogenate tissue was vortexed with 25 μL of internal standard (naringenin 0.4 μg/mL in methanol), 10 μL of methanol and 5 μL HClO_4_ (14%). The mixture was centrifuged for 10 min at 10,000 rpm and 4 °C, and 30 μL of the supernatant was collected and directly injected into the HPLC system.

#### 2.4.3. Total Que Quantification in Serum and Brain Samples

For the total Que determination (parent Que and its metabolite), 15 μL of serum or brain sample and 45 μL of β-glucuronidase enzyme solution (3000 units/mL) were vortexed and incubated for 1 h at 37 °C. After the incubation, 40 μL of the mixture was transferred to a new Eppendorf^®^ tube and mixed with 25 μL of internal standard solution (naringenin 0.4 μg/mL in methanol), 10 μL of methanol and 5 μL HClO_4_ (14%). Centrifugation at 10,000 rpm and 4 °C for 10 min was performed, and 30 μL of the supernatant was collected to be injected into the HPLC system.

### 2.5. HPLC-PDA Method for Que Quantification in Biological Samples

The concentration of Que in both serum and brain tissue samples was measured using the HPLC-PDA Shimadzu prominence system, using naringenin as an internal standard at a concentration of 0.4 μg/mL. This system consists of an LC-20AD Quaternary Gradient Pump with a degasser, an SIL-HT auto-sampler and a photo-diode array detector (SPD-M20A) and is run on the LC Solution^®^ software (LabSolutions, version 1.25 SP4, Kyoto, Japan). The analysis was conducted on a reverse-phase Thermo Scientific™ Aquasil™ C18 column (150 × 4.6 mm, with a particle size of 5 μm) coupled with a C18 precolumn (12.5 × 4.6 mm, with a particle size of 5 μm) of the same type. The mobile phase consisted of water:acetonitrile (65:35 *v*/*v*) ratio adjusted to pH of 2.8 with orthophosphoric acid (80%) and pumped isocratically at a rate of 1 mL/min. The analysis was performed at 25 °C with DAD spectra acquired within the range of 200–400 nm and at a resolution of 4 nm. The run time was 10 min for each injection, and the injection volume was 30 μL. Que and naringenin presented retention times of 6.4 and 8.9 min, respectively. For serum samples, Que and naringenin were detected at 369 nm and 289 nm, respectively, while for brain samples, the detection wavelengths were 256 nm for Que and 289 nm for naringenin, using the SPD-M20A PDA detector from Shimadzu in Kyoto, Japan. The method described by Sanghavi et al. (2014) [38] was optimized for this study, and the calibration curve samples ranged from 0.025 to 0.3 μg/mL of Que. Representative chromatograms are included in the Supplementary materials.

### 2.6. Non-Compartmental Analysis

Phoenix^®^ 8.3.5 (Certara, Princeton, NJ, USA) was used to perform sparse sampling non-compartmental analysis (NCA) of in vivo data. Several pharmacokinetic parameters for serum, brain and olfactory bulb were determined by this methodology, including AUC_0−t_ (the area under the concentration–time curve from time 0 to the last time point of the study), AUCinf (the area under the concentration–time curve extrapolated to infinity), C_max_ (maximum observed concentration) and t_max_ (the time when C_max_ is observed). Additionally, the relative bioavailability of Que following the intranasal (IN) administration of each formulation was calculated. The mean concentration curve data was calculated and combined with subject information to calculate pharmacokinetic parameters and their standard errors (SE). The area under the concentration–time curve (AUC_inf_) was calculated according to the log-linear trapezoidal method with extrapolation to infinity by dividing the last concentration by the terminal slope, λ. The terminal slope was estimated by linear regression analysis on the last four points of the log-transformed concentration vs time plot. The % relative bioavailability (F_rel_) of Que in serum and brain was calculated by comparing the AUCinf after IN and PO administration using Equation (1):

(1) $$\%F_{rel}=\frac{{AUC}_{inf\left(IN\right)}\times {Dose}_{\left(PO\right)}}{{AUC}_{inf\left(PO\right)}\times {Dose}_{\left(IN\right)}}\times 100$$

where AUC_inf_ (IN) and AUC_inf_ (PO) are the area under the concentration vs time curve from 0 extrapolated to infinity after IN and PO administration, respectively. Dose (IN) and Dose (PO) are the respective administered doses. The elimination half-life, t_1/2_, was calculated as t_1/2_ = 0.693/λ, after calculation of the terminal slope, λ. The plasma and brain clearance, CLS and CLB, were calculated as CL_S_ = Dose/AUC_inf_ and CLB = Dose/AUC_inf_, respectively, while both parameters are scaled by 1/F, where F is the absolute bioavailability.

### 2.7. Relative Drug Targeting Efficiency Percentage and NTB Direct Transport Percentage Indexes

In order to assess the extent of direct transport of Que to the brain, the indexes of relative drug targeting efficiency percentage (DTE_rel_) and relative direct transport percentage (DTP_rel_) from the nose to the brain can be employed, as reviewed by Kozlovskaya et al. in 2014 [33]. DTE_rel_ represents the relative exposure of the brain to a drug administered through the nasal route compared to oral administration, as indicated by Equation (2).

(2) $${DTE}_{rel}=\frac{{\left(\frac{{AUC}_{0\to t\;(Brain)}}{{AUC}_{0\to t\;(Serum)}}\right)}_{IN}}{\;\;\;\;\;{\left(\frac{{AUC}_{0\to t\;(Brain)}}{{AUC}_{0\to t\;(Serum)}}\right)}_{PO}}\times 100\;$$

The DTE_rel_ values can vary from 0 to +∞, and values higher than 100 indicate the superiority of NTB for brain targeting over the oral route.

The second index, known as nose-to-brain DTP_rel_, is employed to calculate the percentage of drug that is transferred directly to brain from the nasal cavity, through the olfactory and trigeminal nerves, versus indirect drug delivery via crossing the blood–brain barrier (BBB) (Equation (3)).

(3) $${DTP}_{rel}=\frac{\;\;{\left[{AUC}_{0\to t\;(Brain)}\right]}_{IN}-\left[{AUC}_{0\to t\;(x)}\right]}{\;\;{\left[{AUC}_{0\to t\;(Brain)}\right]}_{IN}}\times 100\;$$

where ${AUC}_{0\to t\;(x)}$ is calculated by Equation (4):

(4) $${AUC}_{0\to t\;(x)}=\frac{\;\;{\left[{AUC}_{0\to t\;(Brain)}\right]}_{PO}}{\;\;{\left[{AUC}_{0\to t\;(Serum)}\right]}_{PO}}\times {\left[{AUC}_{0\to t\;(Serum)}\right]}_{IN}\;$$

DTP_rel_ values can vary from—∞ to 100, but any value equal to zero or lower implies that the drug is delivered to the brain only indirectly, through the BBB.

### 2.8. Statistical Analysis

The experimental data were analyzed using GraphPad Prism 8.0 software package (GraphPad Software). The Shapiro–Wilk was used to assess the normality of the data. A significance level of *p* < 0.05 was chosen, and all tests were two-tailed with 95% confidence intervals. The results are presented as mean ± SEM. Statistical comparisons were performed between all possible pairs of PK profiles and at each time point within the same profile. Outlier detection was conducted using the interquartile range (IQR) method with a step of 1.5 × IQR. No outliers were detected. One-way ANOVA with Bonferroni post hoc test for multiple comparisons was applied, as well as Mann–Whitney nonparametric test between all group pairs. Kruskal–Wallis test was performed to statistically evaluate the differences between the formulations at every time point of the experiment and post hoc Mann–Whitney was performed to detect individual differences.

## 3. Results

### 3.1. Administration of Nasal Formulations

The products received after the previously described [20] lyophilization procedure (Que-Me-β-CD and Que-HP-β-CD) were two light, slightly yellow powders. They were further blended with the spray-dried mannitol/lecithin microparticles produced and characterized in previous work [35]. The Que-Me-β-CD and Que-HP-β-CD contained 11.7% and 9.2% (*w*/*w*) Que, respectively, while the blend formulations thereof were found to contain 8.5% and 6.0% (*w*/*w*) of the flavonoid. IN administration was performed with Aptar’s Unidose Powder system (UDS), an active device delivering powder formulations by insufflation. The UDS was approved by the U.S. FDA in 2019 for the delivery of an intranasal rescue treatment for severe hypoglycemia in individuals with diabetes [39,40]. Specifically designed for drug deposition in the upper part of the human nasal cavity (olfactory region), this device facilitates the NTB delivery of the administered substance. Furthermore, it can be adapted for use on rats, as it comes with a special tip designed for their noses. The amount of lyophilizate or blend insufflated was constrained by the size of the rat’s nose, as well as the powders’ flow properties.

### 3.2. Que HPLC-PDA Assay

Que was quantified in biological samples by HPLC-PDA. A linear relationship was revealed between the peak area ratios (Que peak area/IS peak area) versus the Que nominal concentration over the examined range (0.025–0.3 μg/mL). Overall correlation coefficients (r) of 0.993 (±1.05%, RSD (%)) and 0.997 (±0.240%, RSD (%)) were obtained from the serum and brain sets of the calibration curves, respectively. The lower limit of detection (LLOD) and quantification (LLOQ) of Que in the rat serum were 0.008 and 0.023 μg/mL, respectively, while in the case of the brain tissue, they were 0.013 and 0.025 μg/mL, respectively. In all cases, back calculated concentrations of the calibration working standards were within ±15% of the nominal value and ±20% for the LLOQ.

### 3.3. Oral and IN Administration of Que-Me-β-CD Lyophilizate and Its Blend with Mannitol/Lecithin Microparticles

#### 3.3.1. Serum Pharmacokinetic Data

**Figure 1:**
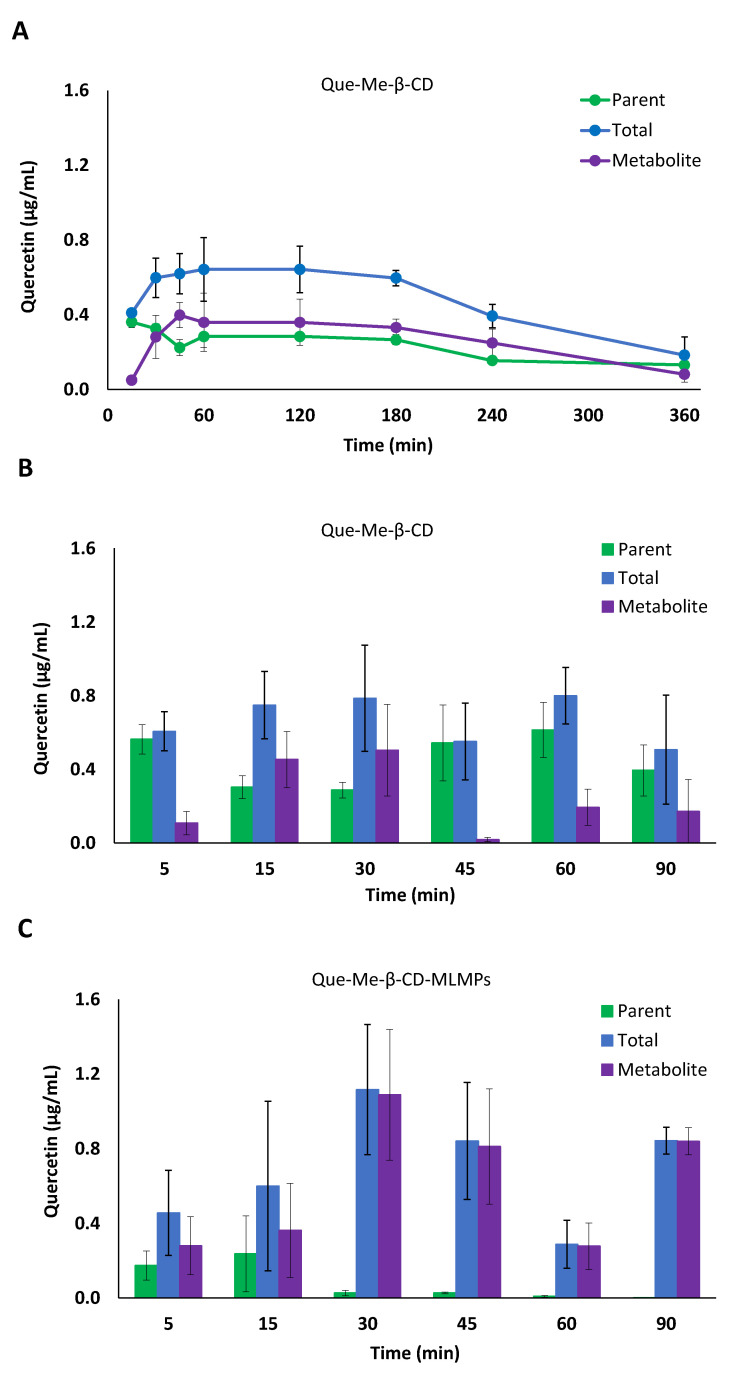
(**A**) Serum concentration–time profiles of the parent (●) and total (●) Que and its metabolite (●) after Que-Me-β-CD lyophilizate PO administration by gavage. (**B**,**C**) Serum levels of parent (■) and total (■) Que and its metabolite (■) after IN administration of (**B**) Que-Me-β-CD lyophilizate and (**C**) Que-Me-β-CD-MLMPs. The data are presented as mean ± SEM, *n* = 3–9.

The serum PK profile of Que-Me-β-CD after its oral administration to the rats revealed a rapid absorption into the bloodstream, resulting in a t_max_ of 15 min and a C_max_ of 0.36 ± 0.03 μg/mL for the parent compound (Table 2). The metabolism of the flavonoid was found to be fast and extensive, as indicated by the levels of metabolite at the same time point (Figure 1A). These levels gradually increased to reach a C_max_ of 0.40 ± 0.07 μg/mL at 45 min after the administration, while the C_max_ (0.64 ± 0.17 μg/mL) of the total Que was reported at 60 min. From 30 min onwards, the metabolite levels remained similar to those of the parent compound (*p* = 0.7130, *n* = 8), resulting in AUC_0−t_ values corresponding to 53% of the total Que AUC_0−t_. The IN administration of the same dose of Que in the Que-Me-β-CD lyophilizate form resulted in a significantly higher C_max_ of the parent compound in serum (0.69 ± 0.15 μg/mL vs 0.36 ± 0.03 μg/mL, *p* = 0.0485, *n* = 4–8) which was achieved 10 min earlier, i.e., at 5 min (Figure 1B, Table 2). The Que metabolism was found to be reduced following the IN administration probably due to the avoidance of the first-pass effect that the nasal route ensures (the metabolite AUC_0−t_ value was equal to 36% of the total Que AUC_0−t_). However, in the case of the Que-Me-β-CD-MLMPs, the metabolism was significantly higher with the metabolite AUC_0−t_ value approaching 91% of the total Que AUC_0−t_ (Figure 1C, Table 2). In the case of the Que metabolite levels, a common trend of a decrease at 45 min followed by an increase at the time point of 90 min was observed for the two IN formulations (Figure 1B,C). The parent Que delivered in the form of the Que-Me-β-CD lyophilizate presented two concentration peaks at 5 and 60 min, respectively, following the same pattern (Figure 1B). Moreover, the IN administration of the MLMP formulation took longer to appear in the bloodstream (t_max_ = 15 min), comparable to the lyophilizate given orally. The blending of the Que-Me-β-CD lyophilizate with the MLMPs had a negative impact on the emitted dose, leading to the administration of a lower amount of Que (0.47 mg vs. 0.88 mg). Even though this dose was half of the one given intranasally with the Que-Me-β-CD, similar values for the AUC_0−t_ and C_max_ of the total Que were reported (*p* = 0.7143, *n* = 3–6, Table 2).

#### 3.3.2. Brain Pharmacokinetic Data

**Figure 2:**
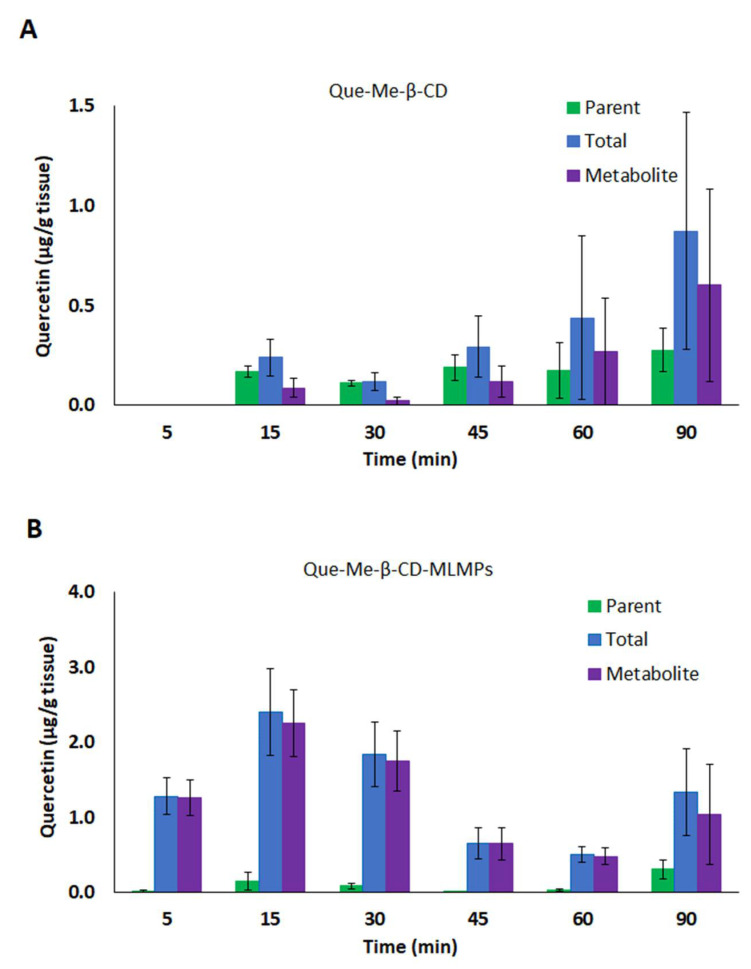
Total brain levels of the parent (■) and total (■) Que and its metabolite (■) after IN administration of (**A**) Que-Me-β-CD lyophilizate and (**B**) Que-Me-β-CD-MLMPs. The data are presented as mean ± SEM, *n* = 3.

**Figure 3:**
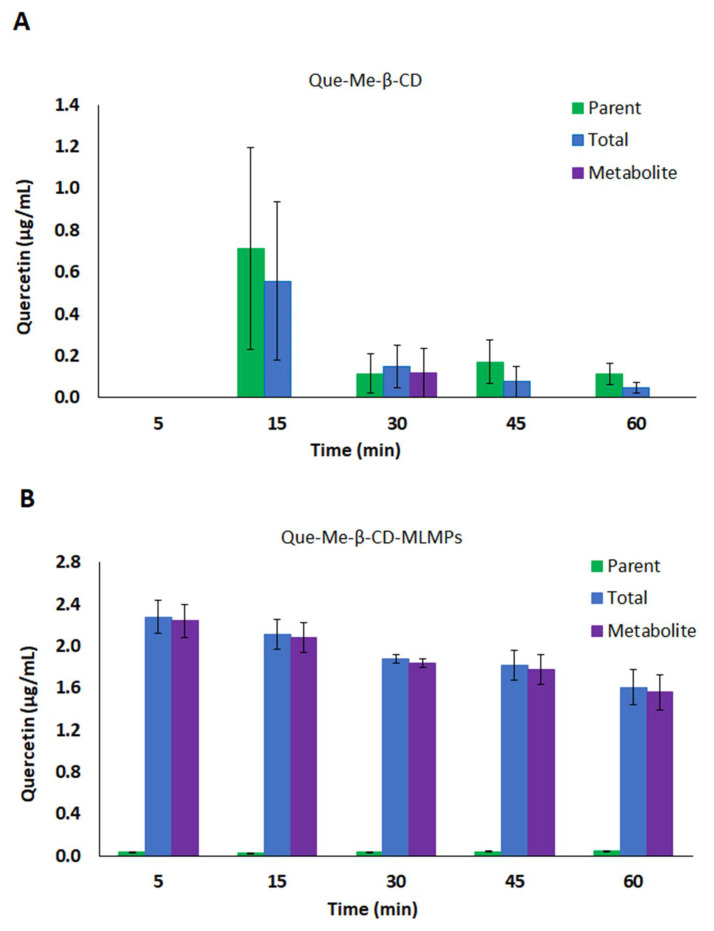
Olfactory bulb levels of the parent (■) and total (■) Que and its metabolite (■) after IN administration of (**A**) Que-Me-β-CD lyophilizate and (**B**) Que-Me-β-CD-MLMPs. The data are presented as mean ± SEM, *n* = 3.

The lower serum AUC_0−t_ values reported in the case of the IN formulations can be explained by more efficient CNS targeting compared to that of the oral administration, which led to undetectable Que levels (of both the parent Que and its metabolite) in the brain tissue (Figure 2, Table 3). In particular, the IN administration of Que-Me-β-CD-MLMPs resulted in an AUC_0−t_ of 108.3 ± 14.64 min × μg/mL for the total Que, with th metabolite levels accounting for a high proportion (92% of the AUC_0−t_ of the total Que) and significantly lower levels of the parent Que (8% of the total AUC_0−t_). The C_max_ of the total Que in the brain was reported 15 min after the administration (2.40 ± 0.572 μg/mL), followed by a second peak at 90 min (1.03 ± 0.670 μg/mL). A higher amount is observed in the form of the metabolite (*p* = 0.0087, *n* = 6), with a C_max_ of 2.25 ± 0.450 μg/mL, while the levels of the parent compound remained consistently low throughout the sampling period after the Que-Me-β-CD-MLMPs administration (Figure 2B). The metabolite’s t_max_ at 15 min indicates the rapid glucuronidation of Que, probably initiated upon the first contact with the nasal environment [41]. The high levels of the total Que (C_max_ of 2.28 ± 0.157 μg/mL) measured in the olfactory bulb (Figure 3) confirm the contribution of the direct NTB delivery, as all the quantified amounts in this neural structure will ultimately reach brain tissue. Since nearly all of the determined amounts in the olfactory bulb from the first time point of 5 min were transformed into the metabolite (97.5 ± 1.2% of the total Que), it can be hypothesized that metabolism begins on the mucosa surface of the nasal cavity [41].

The double-peak phenomenon also described in the serum PK data is more pronounced in the Que-Me-β-CD-MLMPs’ brain profile (0.15 ± 0.12 and 0.31 ± 0.13 μg/mL at 15 and 90 min, respectively), while for the Que-Me-β-CD lyophilizate, the t_max_ is mainly reported at 90 min for both the parent Que and its metabolite, while the peak observed at 15 min is the first measurable brain concentration (Figure 2). Furthermore, in this case, lower brain levels were reported, resulting in an AUC_0−t_ equal to 32.0 ± 13.1 min × μg/mL for the total Que. The contribution of the metabolite was found to be significant in the Que-Me-β-CD lyophilizate as well, accounting for 71% of the AUC_0−t_ of the total Que, while the remaining portion (about 32%) was attributed to the parent Que. It is important to note that the metabolite levels became measurable in the olfactory bulb from the time point of 30 min onwards, indicating that Que is less accessible to mucosa enzymes in the absence of MLMPs (Figure 3). The Que-Me-β-CD-MLMP powder enhanced the brain’s exposure to Que, achieving increases of 65 and 70% in the C_max_ and AUC_0−t_, respectively, considering that half of the dose of the Que-Me-β-CD lyophilizate was given (Figure 2, Table 3).

### 3.4. Oral and IN Administration of Que-HP-β-CD Lyophilizate and Its Blend with Mannitol/Lecithin Microparticles

#### 3.4.1. Serum Pharmacokinetic Data

**Figure 4:**
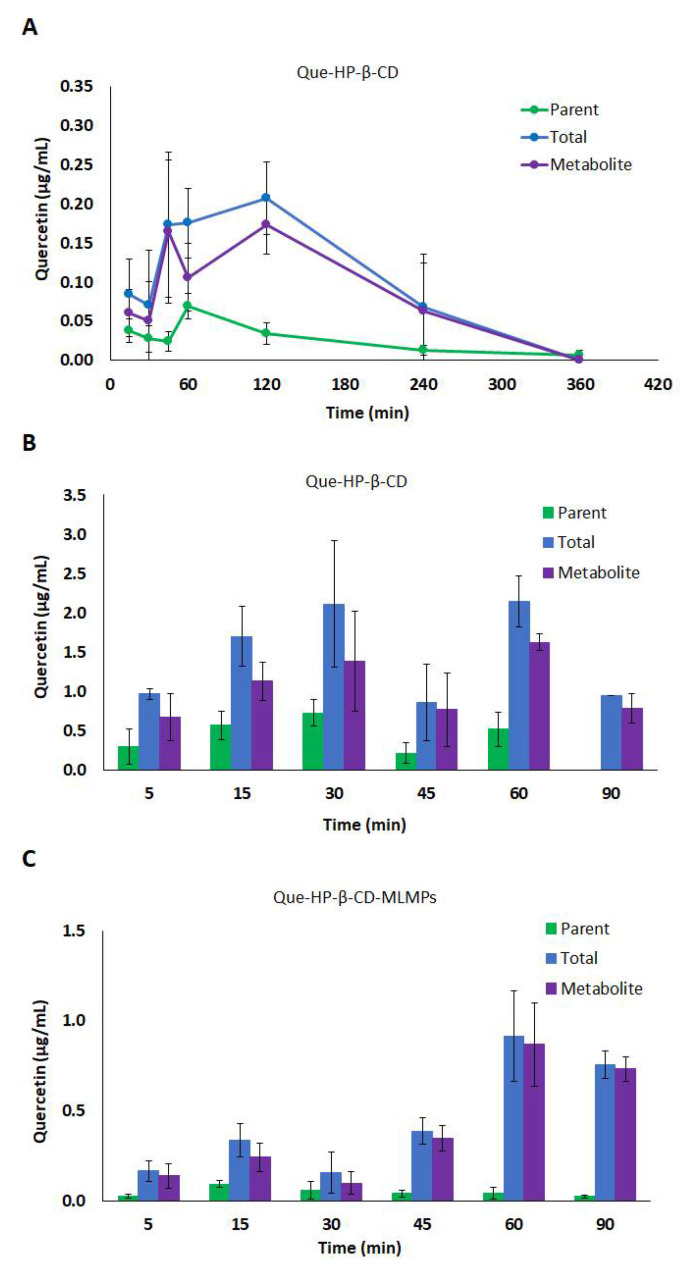
(**A**) Serum concentration–time profiles of the parent (●) and total (●) Que and its metabolite (●) after Que-HP-β-CD lyophilizate PO administration via gavage method. (**B**,**C**) Serum levels of parent (■) and total (■) Que and its metabolite (■) after IN administration of (**B**) Que-HP-β-CD lyophilizate and (**C**) Que-HP-β-CD-MLMPs. The data are presented as mean ± SEM, *n* = 3–4.

Compared to the lyophilizate with methyl-β-cyclodextrin, the orally administered Que-HP-β-CD lyophilizate led to a slower absorption of Que, which resulted in t_max_ values at 60 and 120 min for the parent Que and its metabolite, respectively. The levels of the metabolite were high, with a C_max_ equal to (0.17 ± 0.04 μg/mL), while its AUC_0−t_ corresponded to 80% of the AUC_0−t_ value of the total Que (Figure 4A, Table 4). Accordingly, the levels of the parent Que were significantly lower (C_max_= 0.07 ± 0.02 μg/mL, *p* = 0.0286, *n* = 3) and characterized by faster elimination from the bloodstream (t_1/2_= 99 and 178 min, for the parent Que and its metabolite, respectively). The IN administration of the Que-HP-β-CD lyophilizate resulted in slightly lower levels of the metabolite compared to the oral route (the metabolite’s AUC_0−t_ was equal to 74% of the respective value of the total Que), whereas more extensive metabolism was reported in the case of the blend (the metabolite’s AUC_0−t_ was equal to 91% of the respective value of the total Que) (Table 4). The presence of MLMPs improved the flow properties of the lyophilizate leading to a two-fold higher emitted dose for the blend formulation (the Que doses were equal to 0.15 and 0.34 mg for the Que-HP-β-CD lyophilizate and the blend, respectively). Despite the lower dose, the Que formulated as the lyophilizate with hydroxypropyl-β-cyclodextrin was found to reach the bloodstream more rapidly and efficiently (*p* = 0.0286, *n* = 3), presenting a C_max_ of 2.14 μg/mL at 15 min (total Que), compared to the Que-HP-β-CD-MLMPs blend which needed twice as long (t_max_= 30 min) to reach the C_max_ of 0.91 μg/mL (the total Que). The t_max_ of the metabolite was reported for both IN formulations at 60 min after the administration. In the case of the parent Que and its metabolite levels, a trend of a decrease at 45 min followed by an increase at the time point of 60 min was observed for the Que-HP-β-CD lyophilizate (Figure 4B), which was also reported in the serum PK data of both IN formulations with Me-β-CD.

#### 3.4.2. Brain Pharmacokinetic Data

**Figure 5:**
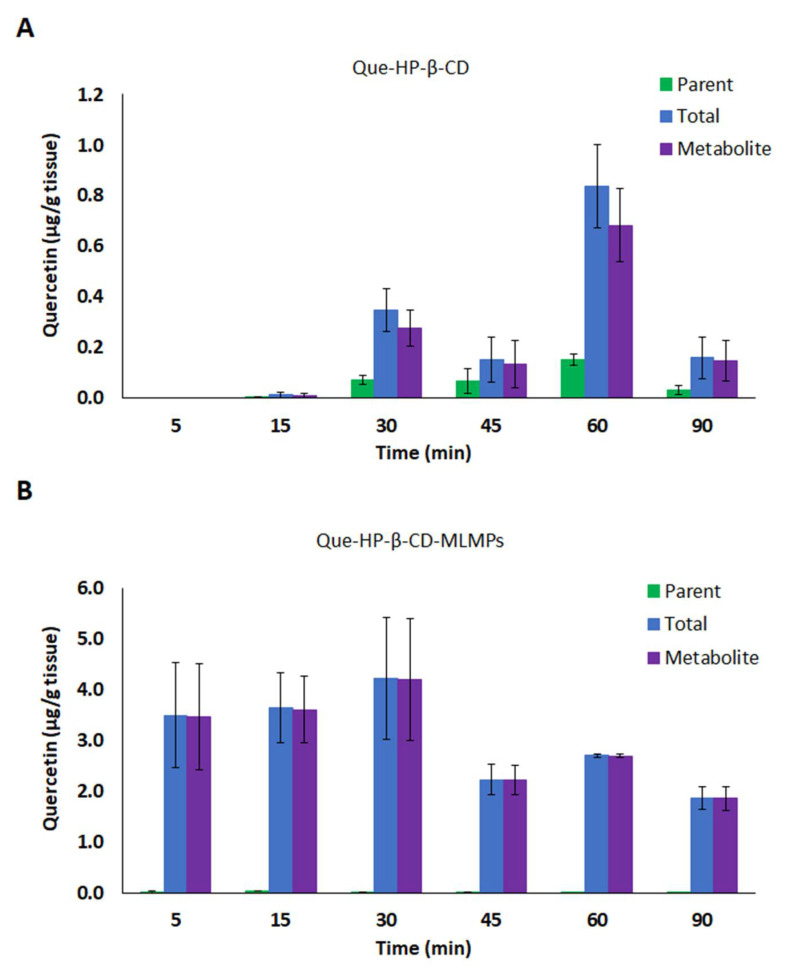
Total brain levels of the parent (■) and total (■) Que and its metabolite (■) after IN administration of (**A**) Que-HP-β-CD lyophilizate and (**B**) Que-HP-β-CD-MLMPs. The data are presented as mean ± SEM, *n* = 3.

**Figure 6:**
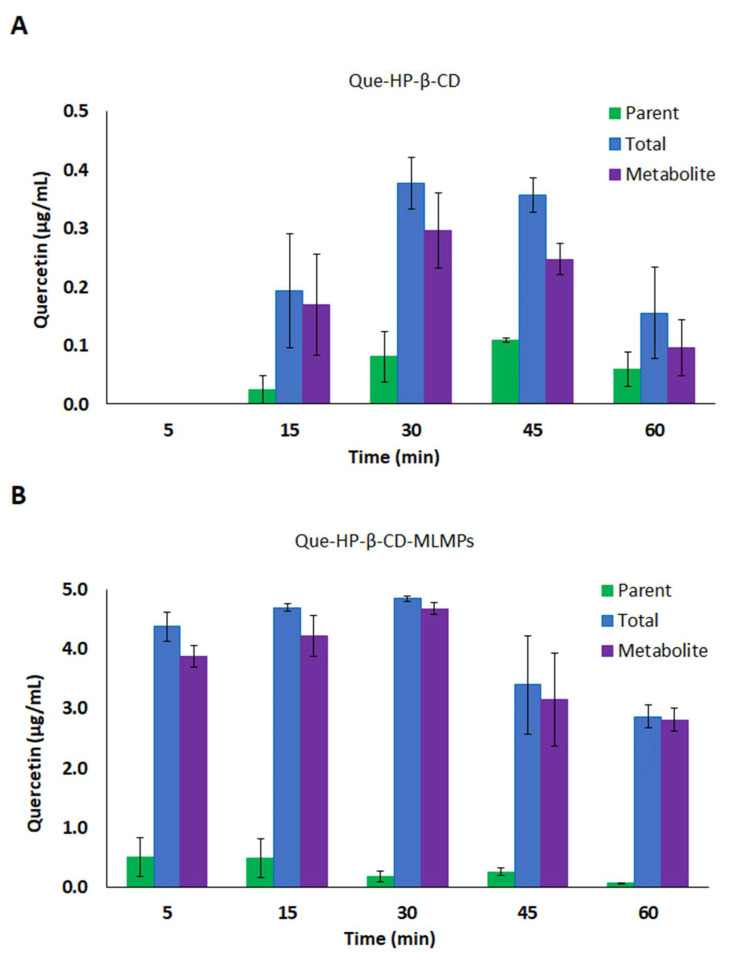
Olfactory levels of the parent (■) and total (■) Que and its metabolite (■) after IN administration of (**A**) Que-HP-β-CD lyophilizate and (**B**) Que-HP-β-CD-MLMPs. The data are presented as mean ± SEM, *n* = 3.

The contribution of the MLMPs to brain delivery was strongly pronounced in the case of Que-HP-β-CD, as revealed by the values for the AUC_0−t_ and C_max_ of the lyophilizate and its blend with the microparticles (Figure 5, Table 5). Specifically, the Que-HP-β-CD-MLMP powder presented an AUC_0−t_ of 257.1 ± 22.4 min·μg/mL, while the C_max_ reached the highest value (4.22 ± 1.21 μg/mL, *p* = 0.0344, *n* = 3) of all the tested IN formulations. Moreover, this formulation allowed for the fast delivery of Que to the brain, mainly in the form of the metabolite, as the transformation to glucuronide occurred in almost all the administered amounts (the metabolite’s AUC_0−t_ was equal to 99.6% of the respective AUC_0−t_ value of the total Que) (Figure 5B). The transformation of nearly all the determined amounts in the olfactory bulb within the first 5 min into metabolites (94.0 ± 7.1% of the total Que) is a common feature among the blends (Figure 3 and Figure 6). The AUC_0−t_ of Que-HP-β-CD in the brain was found to be 79% lower than the respective AUC_0−t_ value in serum, and the high t_max_ (60 min) in the brain possibly indicates a contribution to transport other than direct NTB delivery. Hence, the Que-HP-β-CD lyophilizate is considered more appropriate for Que delivery to the bloodstream. However, it can be detected in low levels in the brain (the C_max_ of total Que was equal to 28.9 ± 5.14 μg/mL) when given intranasally, in contrast with its oral administration, which leads to undetectable levels (of both the parent Que and its metabolite) in brain tissue (Table 5). Moreover, the high levels reported in the olfactory bulb after the IN administration of both HP-β-CD formulations affirm the direct transport of Que through the olfactory pathway directly to the brain tissue (Figure 6). The same pattern of lower and higher Que levels at 45 and 60 min, respectively, noted in the serum PK data after the Que-HP-β-CD lyophilizate administration, was also observed in the brain tissue metabolite concentration for both formulations (Figure 5).

### 3.5. Comparative Profiles of All the Tested Formulations

**Figure 7:**
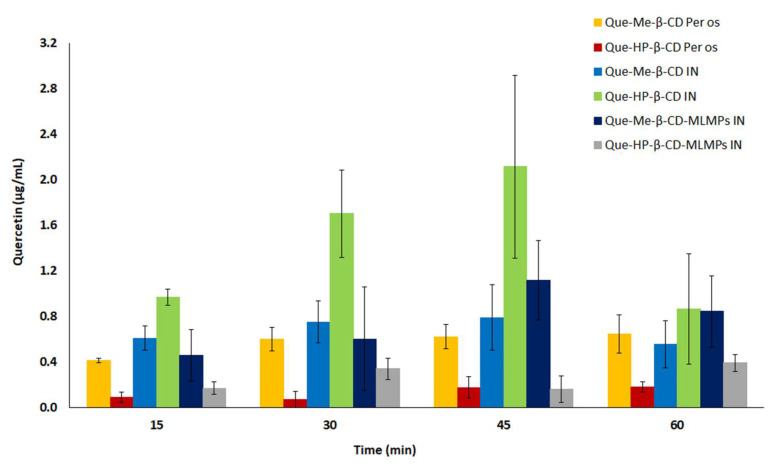
Serum levels of the total Que after Que-Me-β-CD lyophilizate (■) and Que-HP-β-CD lyophilizate (■) PO administration, as well as Que-Me-β-CD lyophilizate (■), Que- HP-β-CD lyophilizate (■), Que-Me-β-CD-MLMPs (■) and Que- HP-β-CD-MLMPs (■) IN administration. The data are presented as mean ± SEM, *n* = 3–9.

The comparative mapping of the total Que serum and brain profiles after oral and IN administration allows for an overall assessment of their performance in brain targeting. The serum levels of Que after the IN administration of the Que-HP-β-CD lyophilizate led to notable fractions of the flavonoid absorbed in the blood (Figure 7), reaching the highest relative bioavailability (F_rel_) compared to that of all the tested formulations and modes of administration, i.e., 391%, 500%, 1362%, 689% and 730% for Que-HP-β-CD (PO), Que-Me-β-CD (PO), Que-Me-β-CD (IN), Que-HP-β-CD-MLMPs (IN) and Que-Me-β-CD-MLMPs (IN), respectively. Furthermore, despite the lower Que levels achieved in the blood after the per os administration of the Que-HP-β-CD lyophilizate (with an AUC_0−t_ of 34.2 ± 6.36 μg/mL vs. 153.0 ± 13.15 μg/mL for the Que-Me-β-CD) due to the lower administered dose, the F_rel_ compared to the Que-Me-β-CD was 128%. As for a dose that is 5.7 times higher, i.e., 0.830 mg vs. 0.145 mg for Que-Me-β-CD and Que-HP-β-CD, respectively (Figure 7), the better performance of Que-HP-β-CD in systemic delivery is revealed from both the IN and per os data. However, the two MLMPs’ formulations did not present significant differences in their ability to deliver Que in the bloodstream, with a F_rel_ of 106% of the Que-HP-β-CD-MLMP lyophilizate compared to the Que-Me-β-CD-MLMPs.

**Figure 8:**
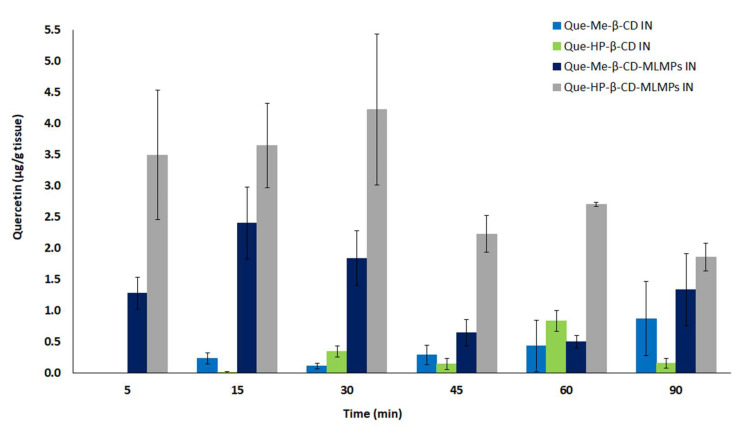
Total brain levels of the total Que after Que-Me-β-CD lyophilizate (■), Que- HP-β-CD lyophilizate (■), Que-Me-β-CD-MLMPs (■) and Que- HP-β-CD-MLMPs (■) IN administration. The data are presented as mean ± SEM, *n* = 3–9.

As depicted in Figure 8, the brain targeting was more efficient using the MLMP formulations. Between the two blends, the Que-HP-β-CD-MLMPs managed to achieve 1.75 and 2.4 times higher C_max_ and AUC_0−t_ values, respectively, compared to the Que-Me-β-CD-MLMPs. Specifically, the IN administration of the Que-HP-β-CD-MLMPs blend showed the highest F_rel_ in the brain compared to all the tested IN formulations, i.e., 385%, 2079%, and 328% compared to the Que-HP-β-CD, Que-Me-β-CD and Que-Me-β-CD-MLMPs, respectively. The values of the brain F_rel_ for each IN formulation compared to the oral administration of the lyophilizates cannot be defined as no measurable brain levels of the Que (of both the parent Que and its metabolite) were observed after oral administration.

The relative % DTE (DTE_rel_) values of all the tested IN formulations cannot be mathematically defined as the brain AUC_0−t_ values after the Que-Me-β-CD and Que-HP-β-CD oral administration were equal to zero. Therefore, the relative % DTP (DTP_rel_) values of the two IN lyophilizates and blends were calculated to be 100%, indicating that Que reached the brain directly from the nasal cavity (via the olfactory and trigeminal nerves), without the contribution of systemic circulation.

## 4. Discussion

The poor absorption and rapid metabolism of Que in the gastrointestinal tract hinder its well-documented therapeutic potential in CNS disorders [42,43,44]. Specifically, the low solubility of the compound and the permeability restrictions of the BBB limit Que’s admission into the brain, necessitating new formulation technologies to enhance its bioavailability [43]. The complexation of Que with Me-β-CD and HP-β-CD has been found to increase the solubility of the flavonoid 6–50 times in a wide range of pHs, from 1.2 to 7.4 [30,31,32]. In particular, at less acidic conditions, i.e., pHs of 6.8 and 7.4, the solubilizing effect of HP-β-CD was superior to that of Me-β-CD, probably due to the formation of a second 1:2 (Que/CD) complex, according to the phase solubility study at a pH of 6.8 [20]. In the context of this PK study, the oral administration of pure Que was not performed due to the low solubility of the compound (0.004–0.016 mg/mL), which made intragastric delivery impossible to an aqueous solution at the required concentration. Hence, the two lyophilizates were orally administered as the reference for evaluating the nasal formulations.

The serum levels after the oral administration of each lyophilizate are in line with the results of Ou-yang et al. (2013) [45], who performed a Que and rutin PK study in rats using a mulberry leaf extract of total flavones. Specifically, the Cmax/dose ratio of 0.42 reported in that study for the parent Que [45] was found to be similar to the respective ratios of Que-Me-β-CD and Que-HP-β-CD after oral administration (0.43 and 0.48, respectively). Nevertheless, in the study by Yang et al. (2016) [46], a ratio of 0.03 was achieved after the intragastric administration of 100 mg/kg of pure Que in rats. Hence, it can be considered that the two lyophilizates can enhance the flavonoid’s delivery into the bloodstream, taking advantage of their cyclodextrin-dependent solubilizing effect on Que in gastric fluids. In the same study, the single oral administration of Que did not reveal measurable levels in the brain [46]. However, a 6-week treatment of mice with Que-enriched diets (2 mg/g diet) can lead to low brain levels of 0.085 μg/g [47]. As the oral doses of lyophilizates were selected to be equal to the intranasal ones, they were substantially lower (2.5 and 0.45 mg/kg, for Que-Me-β-CD and Que-HP-β-CD, respectively) resulting in undetectable levels of the parent Que and its metabolite in the brain. To this end, the nasal route constitutes a promising approach to surmount the challenge of low bioavailability, requiring the application of equal or smaller doses to achieve substantial levels in the CNS [48].

The high permeability of the nasal mucosa along with its extensive vascularization enable effective systemic absorption, particularly from the respiratory area, which serves as the main site for systemic entry [49]. The comparative profile of all the tested formulations (Figure 7) demonstrates that the deposition of the Que-HP-β-CD lyophilizate for systemic absorption was more efficient. In particular, a more localized deposition into the respiratory zone is likely responsible for the significant serum bioavailability reported in this case [50]. Moreover, the higher solubilizing effect of HP-β-CD compared to that of Me-β-CD, as documented in a previous solubility study [31,32], allows for a significant fraction of the dose to be available for systemic absorption. In contrast, the blending of lyophilizates with MLMPs can modify their flow properties, leading them to deposit in the upper part of the human nasal cavity (olfactory region), as indicated by the high Que levels in the olfactory bulb (Figure 3 and Figure 6). This alteration facilitates the NTB transport of the compound [50]. The observed double-peak phenomenon in both the serum and brain levels after IN administration can be attributed to the release pattern of Que from CD complexes and the longer time required for the uncomplexed Que to be solubilized in the limited volume of nasal fluids.

Mannitol/lecithin microparticles, originally proposed by Balducci et al. (2013) [35] to embed desmopressin for systemic absorption from the nasal cavity, are also considered suitable for nasal delivery because of their agglomerating properties [51]. Alone or blended with a second microparticle population, they form coarser free-flowing agglomerates that can favor deposition at the nasal cavity and minimize the risk of inhalation into the lungs. Here, when the MLMPs were blended with the Que-CD lyophilizate powders, they spontaneously aggregated on the flake surfaces of the lyophilizates. Both the lyophilizates and the blend formulations quickly dissolved upon contact with the nasal mucosa fluids. After comparing their performance in vitro and ex vivo, it was understood that the HP-β-CD formulations performed better in terms of their diffusion and permeation profile [32]. Additionally, blending lyophilizates with hydrophilic microparticles, consisting of mannitol (logP = −3.4) and a small amount of lecithin, can enhance the wettability of the covered flakes. This facilitates the disintegration of their combined structure and the release of Que into the nasal mucosa [52]. However, soybean lecithin is a highly lipophilic molecule (logP = 9.1) capable of forming inclusion complexes with β-CD [53]. Therefore, it can be hypothesized that lecithin molecules can replace the less lipophilic Que in the hydrophobic cavity of the CD cone upon contact with nasal fluids. This hypothesis is based on a documented correlation between the lipophilicity of the guest molecule and the binding constant of the complex [54]. The faster and more extensive transformation of Que into glucuronide in the cases of blend formulations can be explained following this assumption, as the Que molecules become more exposed to the UDP-glucuronosyltransferases (UGTs) of the nasal mucosa. UGTs constitute major enzymes of the olfactory area, being involved in several processes such as odorant clearance and the protection of the respiratory system [41].

Among all the PK profiles obtained from oral and IN administration, a common feature is the presence of high metabolite levels in both serum and brain samples. It is well established that Que glucuronide is the major metabolite of the parent flavonoid, comprising over 90% of the total Que in plasma, along with the sulfate conjugates of isorhamnetin [17]. These metabolites have been reported to exhibit bioactivity, contributing to the antioxidant and anti-inflammatory effects of the parent Que [15,16]. Specifically, the quercetin-3-O-glucoronide can enhance neuroplasticity mechanisms in the brain and express a significant inhibitory effect on Aβ accumulation [18].

Significant concentrations of parent Que and its metabolite were measured in the olfactory bulb (Figure 3 and Figure 6) following the intranasal insufflation of the blends. This direct connection between the bulb and the nasal cavity indicates that the flavonoid is transported directly through the NTB pathways [55]. The notably high concentration in the olfactory bulb holds promise for the targeted brain delivery of Que for Alzheimer’s disease. Olfactory impairment, caused by morphological and signaling changes in the olfactory nerve, is recognized as an early marker of the most common neurodegenerative disorders, such as Alzheimer’s and Parkinson’s disease (PD) [56]. As the % DTE_rel_ value cannot be defined due to the zero levels of Que in brain following the oral administration of lyophilizates, it can be assumed that the levels observed in the cases of IN formulations are only attributable to direct NTB transport (via olfactory and trigeminal nerves). The potential ingestion of a quantity of the formulation is not considered to contribute to these levels. This hypothesis is also evident from the % DTP_rel_ values which indicate that the fraction of the administered dose measured in the brain entered the tissue following the passages of the neuronal connection between the nose and the CNS.

## 5. Conclusions

In the present study, it was demonstrated that the IN administration of Que complexed with either the methylated or the hydroxy propylated β-CD derivative in the form of lyophilizates can lead to significant Que levels in the serum and brain. Moreover, the blending of the lyophilizates with MLMPs formed nasal powders with improved properties in terms of brain targeting. The more efficient transport of Que into the CNS when administered in a blend formulation highlights their applicability in NTB as a possible early treatment against neurodegenerative implications associated with AD, PD or other neurological disorders. Conversely, the more extensive absorption of Que into the bloodstream after the lyophilizate’s nasal administration may encourage researchers to consider these formulations when an enhancement of systemic activity is required.

## Figures and Tables

**Table 1:** Dosing protocol for animal experiments.

Group	Formulation	Mode of Administration	Dose	
			mg/kg	mg
a	Que-Me-β-CD lyophilizate	Per os	2.5	0.83
b	Que-HP-β-CD lyophilizate	Per os	0.45	0.145
c	Que-Me-β-CD lyophilizate	Intranasal	2.7	0.88
d	Que-HP-β-CD lyophilizate	Intranasal	0.45	0.147
e	Que-Me-β-CD:MLMPs (75:25)	Intranasal	1.46	0.47
f	Que-HP-β-CD:MLMPs (75:25)	Intranasal	1	0.34

**Table 2:** Non-compartmental analysis on serum data of Que-Me-β-CD lyophilizate and its blend, using sparse data methodology (*n* = 3).

QUE NCA Serum Pharmacokinetics									
Pharmacokinetic Parameter Value (SE)									
	Nasal Administration						Per os Administration		
Pharmacokinetic Parameter	Que-Me-β-CD Lyophilizate			Que-Me-β-CD-MLMPs			Que-Me-β-CD		
	Parent	Total	Metabolite	Parent	Total	Metabolite	Parent	Total	Metabolite
**AUC_0−t_ (** **min** ** × μg)/mL**	41.2 (5.25)	59.6 (8.12)	21.3 (5.42)	5.4 (2.6)	59.4 (9.70)	54.0 (8.33)	74.6 (4.04)	153 (13.2)	81.2 (12.3)
**C_max_ (μg/mL)**	0.69 (0.15)	0.80 (0.15)	0.50 (0.25)	0.23 (0.20)	1.1 (0.34)	1.1 (0.35)	0.36 (0.031)	0.64 (0.17)	0.40 (0.067)
**AUC_inf_ (min** ** × μg)/mL**	89.3	-	-	5.42	-	-	119	193	96.8
**AUC % Extrapolation**	54	-	-	<1	-	-	37	21	16
**t_max_ (min)**	5/60	60	30/90	15	30	30/90	15	60	45
**t_1/2_ (min)**	85	-	-	13	-	-	235	152	134
**k_el_ (1/min)**	0.008	-	-	0.054	-	-	0.003	0.005	0.005

**Table 3:** Non-compartmental analysis on brain data of Que-Me-β-CD lyophilizate and its blend, using sparse data methodology (*n* = 3).

QUE NCA Brain Pharmacokinetics									
Pharmacokinetic Parameter Value (SE)									
	Nasal Administration						Per os Administration		
Pharmacokinetic Parameter	Que-Me-β-CD Lyophilizate			Que-Me-β-CD-MLMPs			Que-Me-β-CD		
	Parent	Total	Metabolite	Parent	Total	Metabolite	Parent	Total	Metabolite
**AUC_0−t_ (** **min** ** × μg)/g**	14.7 (3.66)	32.0 (13.1)	18.3 (9.51)	8.6 (2.5)	108 (14.6)	99.8 (14.6)	-	-	-
**C_max_ (μg/g)**	0.27 (0.10)	0.84 (0.59)	0.60 (0.48)	0.31 (0.13)	2.4 (0.57)	2.3 (0.45)	-	-	-
**AUC_inf_ (min** ** × μg)/g**	-	-	-	-	-	-	-	-	-
**AUC % Extrapolation**	-	-	-	-	-	-	-	-	-
**t_max_ (min)**	90	90	90	90	15	15	-	-	-
**t_1/2_ (min)**	-	-	-	-	-	-	-	-	-
**k_el_ (1/min)**	-	-	-	-	-	-	-	-	-

**Table 4:** Non-compartmental analysis of serum data of Que-HP-β-CD lyophilizate and its blend, using sparse data methodology (*n* = 3).

QUE NCA Serum Pharmacokinetics									
Pharmacokinetic Parameter Value (SE)									
	Nasal Administration						Per os Administration		
Pharmacokinetic Parameter	Que-HP-β-CD Lyophilizate			Que-HP-β-CD-MLMPs			Que-HP-β-CD Lyophilizate		
	Parent	Total	Metabolite	Parent	Total	Metabolite	Parent	Total	Metabolite
**AUC_0−t_ (** **min** ** × ** **μg)/mL**	37.6 (6.53)	136 (14.8)	99.8 (10.8)	4.2 (0.012)	45.5 (6.27)	41.3 (5.67)	8.9 (1.7)	34.2 (6.36)	27.5 (5.50)
**C_max_ (μg/mL)**	0.73 (0.20)	2.2 (0.37)	1.6 (0.19)	0.094 (0.018)	0.91 (0.25)	0.87 (0.25)	0.07 (0.02)	0.21 (0.47)	0.17 (0.037)
**AUC_inf_ (min** ** × ** **μg)/mL**	46.6	-	-	6.1	-	-	9.8	45.7	43.9
**AUC % Extrapolation**	19	-	-	30	-	-	9	25	37
**t_max_ (min)**	30/60	30/60	30/60	15	60	60	60	120	120
**t_1/2_ (min)**	37	-	-	52	-	-	99	118	178
**k_el_ (1/min)**	0.019	-	-	0.013	-	-	0.007	0.006	0.004

**Table 5:** Non-compartmental analysis of brain data of Que-HP-β-CD lyophilizate and its blend, using sparse data methodology (*n* = 3).

QUE NCA Brain Pharmacokinetics									
Pharmacokinetic Parameter Value (SE)									
	Nasal Administration						Per os Administration		
Pharmacokinetic Parameter	Que-HP-β-CD Lyophilizate			Que-HP-β-CD-MLMPs			Que-HP-β-CD Lyophilizate		
	Parent	Total	Metabolite	Parent	Total	Metabolite	Parent	Total	Metabolite
**AUC_0−t_ (** **min** ** × μg)/g**	6.0 (1.0)	28.9 (5.14)	23.9 (4.54)	1.0 (0.4)	257 (22.4)	256 (22.2)	-	-	-
**C_max_ (μg/g)**	0.15 (0.026)	0.84 (0.20)	0.68 (0.18)	0.039 (0.031)	4.2 (1.2)	4.2 (1.2)	-	-	-
**AUC_inf_ (min** ** × μg)/g**	-	-	-	1.1	421.6	421.2	-	-	-
**AUC % Extrapolation**	-	-	-	3	39	39	-	-	-
**t_max_ (min)**	60	60	60	15	30	30	-	-	-
**t_1/2_ (min)**	-	-	-	17	61	62	-	-	-
**k_el_ (1/min)**	-	-	-	0.041	0.011	0.011	-	-	-

## Data Availability

The data presented in this study are available in the present article.

## Supplementary Materials

The following supporting information can be downloaded at: https://www.mdpi.com/article/10.3390/pharmaceutics15082036/s1, Figure S1. Representative chromatogram of blank serum sample (λ = 369 nm). Figure S2. Representative chromatogram of blank brain sample (λ = 369 nm). Figure S3. Representative chromatogram of ISTD (Naringenin, 0.4 μg/mL) sample spiked in blank serum (λ = 289 nm, *t* = 8.9 min). Figure S4. Representative chromatogram of ISTD (Naringenin, 0.4 μg/mL) sample spiked in blank brain (λ = 289 nm, *t* = 8.9 min). Figure S5. Representative chromatogram of calibration curve sample (Quercetin, 0.125 μg/mL) sample spiked in blank serum (λ = 369 nm, *t* = 6.4 min). Figure S6. Representative chromatogram of calibration curve sample (Quercetin, 0.125 μg/mL) sample spiked in blank brain (λ = 369 nm, *t* = 6.4 min).
