# Supplementary material for: Comparative Serum and Brain Pharmacokinetics of Quercetin after Oral and Nasal Administration to Rats as Lyophilized Complexes with β-Cyclodextrin Derivatives and Their Blends with Mannitol/Lecithin Microparticles
Source: Pharmaceutics. 2023 Jul 28;15(8):2036. doi: 10.3390/pharmaceutics15082036 (PMC10459069; doi:10.3390/pharmaceutics15082036)
Supplement: Supplementary file 1 [file pharmaceutics-15-02036-s001.zip › pharmaceutics-2497774-supplementary.pdf]

Supplementary Material

Title: Comparative serum and brain pharmacokinetics of quercetin after oral and nasal administration to rats as lyophilized complexes with β-cyclodextrin derivatives and their blends with mannitol/lecithin microparticles

Konstantina Manta[1,†], Paraskevi Papakyriakopoulou[1,†], Anna Nikolidaki[1], Evangelos Balafas[2], Nikolaos Kostomitsopoulos[2], Sabrina Banella[3], Gaia Colombo[3], Georgia Valsami[1,*]

1. Department of Pharmacy, School of Health Sciences, National and Kapodistrian University of Athens, 15784, Greece
2. Laboratory Animal Facility, Centre of Clinical, Experimental Surgery and Translational Research, Biomedical Research Foundation of the Academy of Athens, 11527, Athens, Greece
3. Department of Life Sciences and Biotechnology, University of Ferrara, 44121, Italy

*Corresponding author: valsami@pharm.uoa.gr

HPLC-PDA chromatograms for Que quantification in biological samples

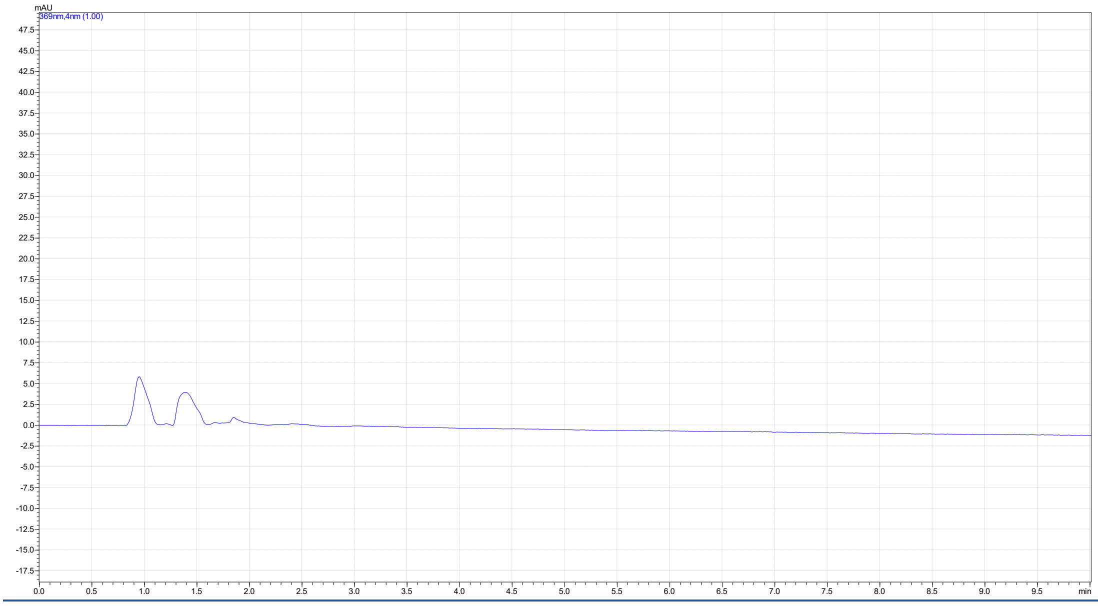

Figure S1. Representative chromatogram of blank serum sample (λ=369 nm).

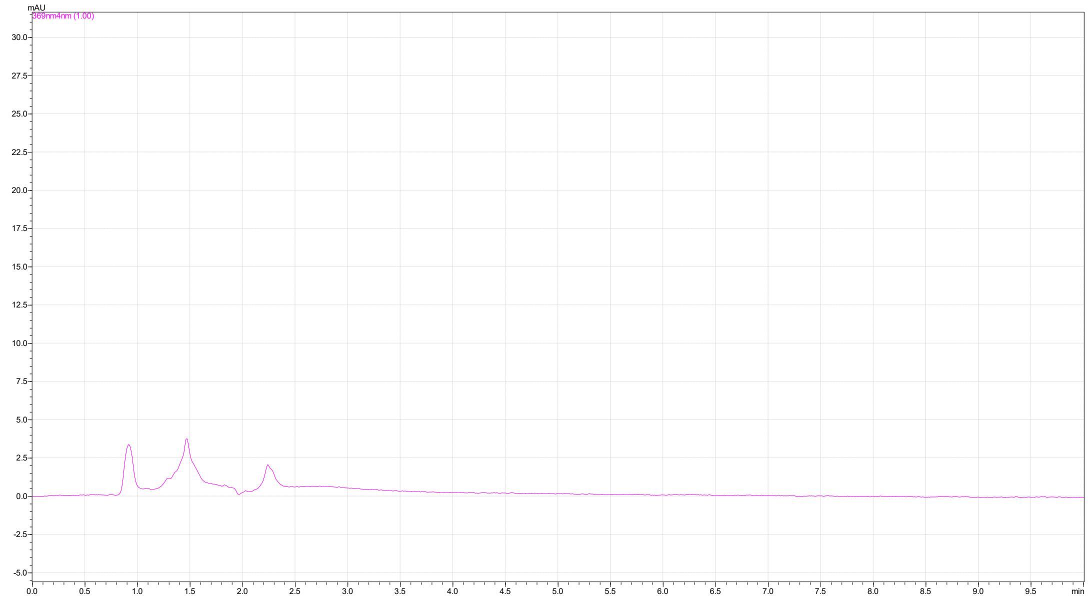

Figure S2. Representative chromatogram of blank brain sample (λ=369 nm).

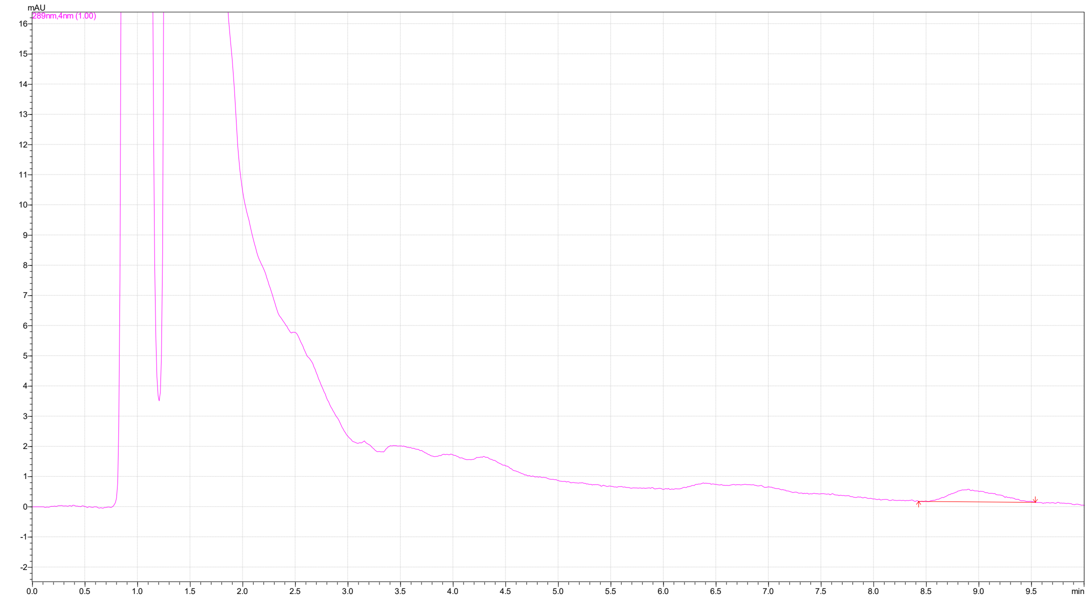

Figure S3. Representative chromatogram of ISTD (Naringenin, 0.4 μg/mL) sample spiked in blank serum (λ=289 nm, t= 8.9 min).

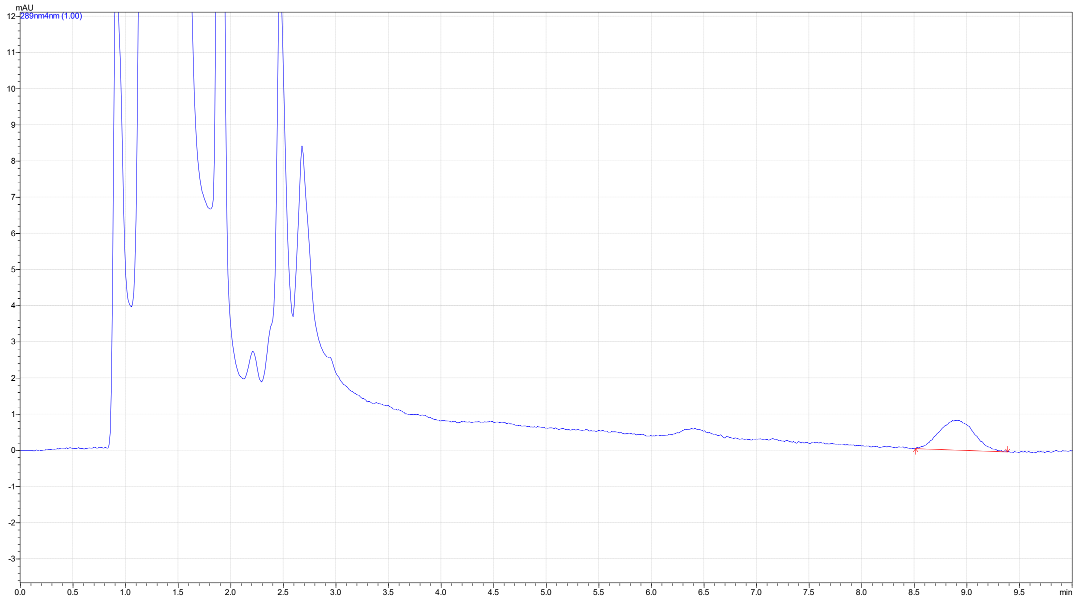

Figure S4. Representative chromatogram of ISTD (Naringenin, 0.4 µg/mL) sample spiked in blank brain (λ=289 nm, t= 8.9 min).

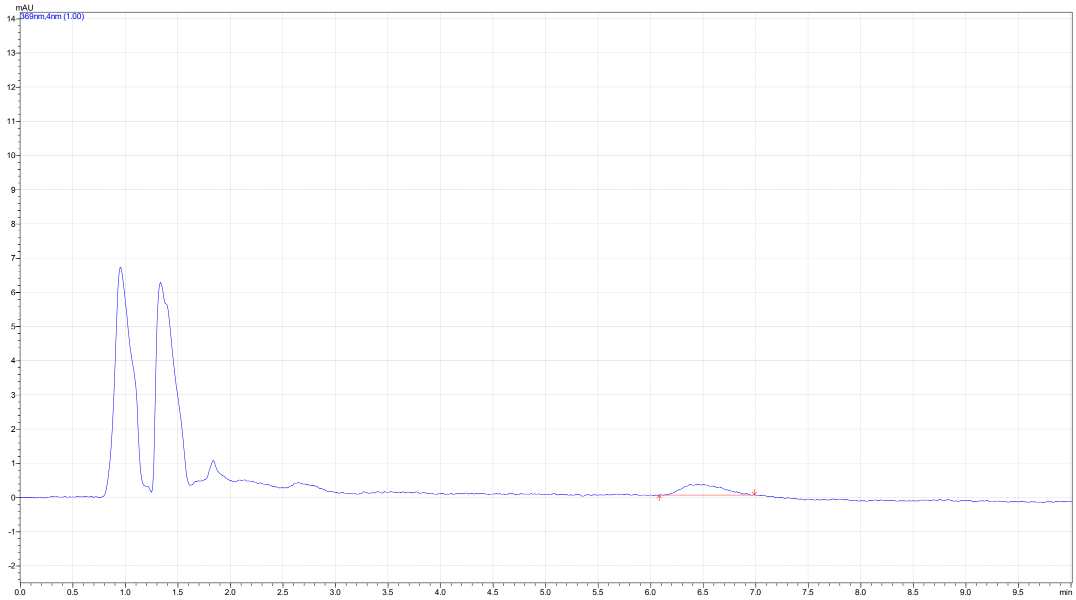

Figure S5. Representative chromatogram of calibration curve sample (Quercetin, 0.125 µg/mL) sample spiked in blank serum (λ=369 nm, t= 6.4 min).

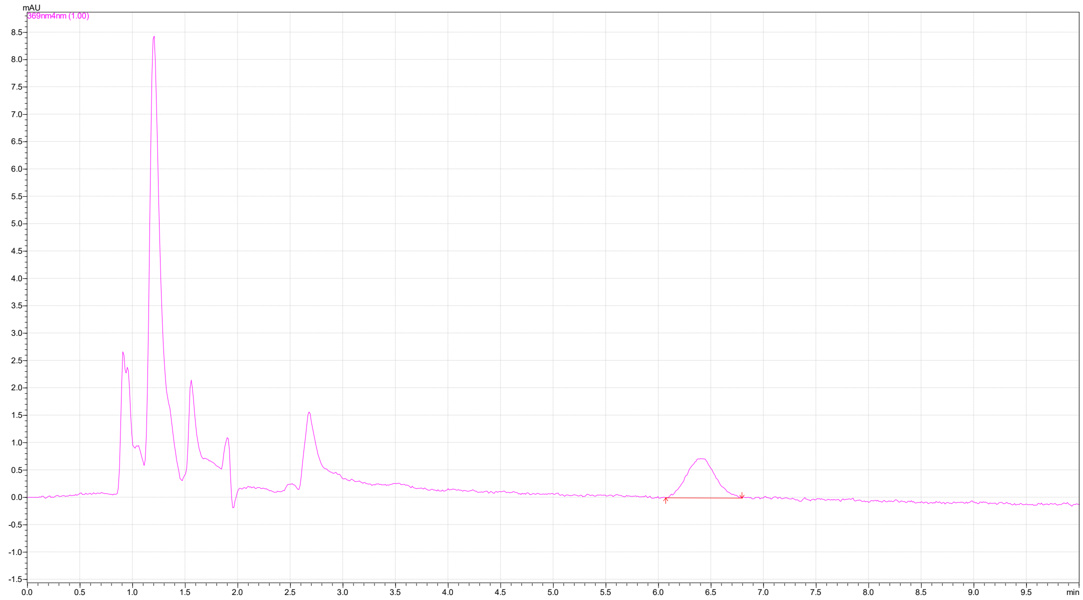

Figure S6. Representative chromatogram of calibration curve sample (Quercetin, 0.125 µg/mL) sample spiked in blank brain (λ=369 nm, t= 6.4 min).
